# Report of a Case Resembling Dry Gangrene, in Which Amputation of the Thigh Was Performed

**Published:** 1830

**Authors:** William Lindsey

**Affiliations:** Richmond, Indiana


### ART. III.—*Report of a case resembling Dry Gangrene, in which amputation of the thigh was performed by* WILLIAM LINDSEY, M. D. of Richmond, Indiana.

The patient Lucinda Dunkin, about 17, emigrated from North Carolina in the early part of last fall. On her journey she was exposed to great fatigue and hardship travelling the greater part of the way on foot, wading the water courses when they were passable in this way, and "camping out" by night. On her arrival in this place she complained of general lassitude and debility, but continued to pursue her usual avocations for some weeks, when she went to Cincinnati to seek employment as a domestic servant. From this time her health gradually became worse until she was confined to her bed, and she continued in that city for three weeks without receiving any medical assistance. At the expiration of this time she again returned to this place, and on her journey, when so unwell as to be unable to sit up, she was exposed in a very inclement season of almost continual rain, very badly clothed, to sleep in the waggon four days, her clothes scarcely dry at any period of this time. She remained for some weeks, the precise time I cannot state, without any regular professional attendance, and in the mean time was put under a steam course by an empiric of the *Steam or Indian Faculty.*

As the patient was very poor and friendless and perfectly destitute of all the necessaries of life, she was "put upon the county," and my good friend Dr. Prichett of Centreville was employed to attend her.

What her disease was during the first five or six weeks of her illness, it is now impossible to determine; but it was doubtless occasioned by the long continued application of cold, and aggravated by renewed exposure. Dr. Prichett found her labouring under a low typhoid fever, that continued to grow worse for two or three weeks, until she was reduced to the very borders of the grave. At length, however, a crisis apparently formed, and the patient appeared to be very slowly convalescing, but in about ten days she was seized with a severe pain in the left foot and leg extending up to the knee, which was followed in a few hours by leaden coloured spots gradually changing to a dark olive, variously interspersed from the knee to the toes. In a few days nature appeared to be drawing a line of separation at the knee joint, producing a solution of continuity followed by sphacelation and sloughing, and in about three weeks from the commencement of the pain the separation was nearly effected. In addition to the ulcer at the knee, three sinuses had been formed, upon the external, internal, and anterior part of the thigh, the two former extending nearly to the upper extremity of the os femoris.

On the 13th of January I was called in to consult on the propriety of an operation. Until within a few days, the extreme debility had forbid any hope of success from this measure, but at present, her general health was improving, and the sinuses had nearly disappeared under a course of general tonics, and the local application of the roller with the astringent and stimulating and injections.

As we considered that the patient must eventually sink under the copious and offensive discharge from the knee, amputation was determined on as a last resort, and the limb was removed by the flap operation, about seven inches above the knee.

Some time previous to the operation, a small ulcer had taken place upon the loins, which from the great weakness of the patient could receive but little attention, until the condition of the stump permitted her to change her position of recumbency, from the back to the right side. By this time the ulcer had become very formidable, measuring three inches in diameter, while a number of small sinuses opening under its margin indicated the general loss of tonicity in the neighboring parts; and when the sloughing was arrested, two of the spinous processes were so far denuded as to be visible at a considerable distance.

After changing her position, two other troublesome ulcers made their appearance, one upon the trochanter major, and the other a little above that process.

The ulcerative process which accompanied these ulcers were attended with some striking peculiarities. The first thing which arrested our attention was a slight discoloration of the surface, unaccompanied by swelling or inflammation, confined to a small point, of a dusky leaden hue similar to that of ecchymosis, gradually changing through the intermediate shades to a deep black, and gradually extending until arrested by stimulating applications; and when arrived at this state, the skin presented to the sense of touch a sensation similar to that produced by rubbing a dry buck skin cushion. In order to produce a separation of the hardened integuments, the most powerful stimulants and vesicatories were necessary, such as tinct Lyttæ, ol Terebin, &c. and after this they healed more kindly under a dressing of Ung, spermaceti covered with a poultice than with any other dressing we applied. After the operation, the health of the patient improved under the use of Sulph. quina and other tonics, with the occasional use of diffusible stimulants and anodynes to meet particular symptoms, and in three weeks the stump was perfectly healed.

By this time however, the ulcers on the loins had become so troublesome as to interfere very materially with the general health, and in a few days after the acatirzation of the stump, a general edema took place over the body, the right leg became enormously distended, and the stump ulcerated afresh. The strength however was supported with tonics stimulants, to the edematous limb were applied a roller, the tinct. of capsicum, and eventually, what served a still better purpose as a dressing, both to this the ulcerated stump, the vene. terebin. This last remedy in reducing the pain and edematous swelling had a most astonishing effect. On examination of the limb after its removal, the peritonteum generally was found dry and detached; many parts of it presented a dry, shrunken and crisped appearance; the whole foot was so divested of moisture that I was enabled without any difficulty to preserve it, with the leg some distance above the ancle. The teguments having been removed by stimulating dressing the whole upper surface presented a fine exhibition of the more superficial vascular distribution; the blood preserving its natural colour so as beautifully to represent a fine red injection.

The knee joint was very much diseased: the head of the fibula was separated, and the head of the femoris and tibia were carious to the distance of two or three inches from the joint.

*Remarks by the Editor.*—The details of this interesting case exhibit a degree of skill and humanity on the part of the physicians who attended this unfortunate patient, that is highly creditable to the profession.

To the greater part of the world, the reward which follows acts of disinterested benevolence is so remote, that such occurrences cannot be too highly valued. We have seen a limb that had been amputated for dry gangrene by Dr. Gibson, but which differed from the case described by Dr. L. in several important particulars. The disease commenced in the foot and proceeded up the leg, as if by continuous sympathy, until it was arrested by amputation, and the amputated limb presented the appearance of a well dried preparation covered with varnished leather, without exhibiting on its surface any trace of organization. In the present case, the disease appears to have been confined to the skin and cellular membrane, and to have depended upon constitutional derangement.

Since the above was written we are informed by Dr. Lindsey that the patient has recovered her health perfectly.

